# Proactive Recruitment of Frontoparietal and Salience Networks for Voluntary Decisions

**DOI:** 10.3389/fnhum.2017.00610

**Published:** 2017-12-12

**Authors:** Natalie Rens, Stefan Bode, Hana Burianová, Ross Cunnington

**Affiliations:** ^1^Queensland Brain Institute, The University of Queensland, Brisbane, QLD, Australia; ^2^Melbourne School of Psychological Sciences, The University of Melbourne, Melbourne, VIC, Australia; ^3^Department of Psychology, University of Cologne, Cologne, Germany; ^4^Centre for Advanced Imaging, The University of Queensland, Brisbane, QLD, Australia; ^5^Department of Psychology, Swansea University, Swansea, United Kingdom; ^6^School of Psychology, The University of Queensland, Brisbane, QLD, Australia

**Keywords:** free choice, self-initiated decision, action selection, partial least squares, multi-voxel pattern analysis, eye-tracking

## Abstract

There is evidence that neural patterns are predictive of voluntary decisions, but findings come from paradigms that have typically required participants to make arbitrary choices decisions in highly abstract experimental tasks. It remains to be seen whether proactive neural activity reflects upcoming choices for individuals performing decisions in more complex, dynamic, scenarios. In this functional Magnetic Resonance Imaging (fMRI) study, we investigated proactive neural activity for voluntary decisions compared with instructed decisions in a virtual environment, which more closely mimicked a real-world decision. Using partial least squares (PLS) analysis, we found that the frontoparietal and salience networks were associated with voluntary choice selection from a time at which decisions were abstract and preceded external stimuli. Using multi-voxel pattern analysis (MVPA), we showed that participants’ choices, which were decodable from motor and visual cortices, could be predicted with lower accuracy for voluntary decisions than for instructed decisions. This corresponded to eye-tracking data showing that participants made a greater number of fixations to alternative options during voluntary choices, which might have resulted in less stable choice representations. These findings suggest that voluntary decisions engage proactive choice selection, and that upcoming choices are encoded in neural representations even while individuals continue to consider their options in the environment.

## Introduction

Voluntary, or free, decisions are defined by the ability to select an option from amongst a number of available alternatives (Haggard, 2005; Thimm et al., 2012). Typically, voluntary decisions have been contrasted with instructed decisions at the time the decision is made, emphasizing differences in self-initiating a response (Cunnington et al., 2002; Rowe et al., 2005; Forstmann et al., 2006; Taylor et al., 2008; Dominguez et al., 2011; Krieghoff et al., 2011; Thimm et al., 2012). However, decision-making is increasingly viewed as a proactive process in which neural activity prior to the decision execution corresponds to preparation of the upcoming choice (Friston, 2010; Pezzulo and Ognibene, 2012). Previous studies have shown the ability to predict voluntary choices in highly simplified contexts, for example making decisions between left and right buttons without any environment in which these decisions would have an effect (e.g., Soon et al., 2008; Bode et al., 2011), between abstract decision to add or subtract two numbers without any consequence (e.g., Soon et al., 2013), or between object categories that again lacked any context to give these decisions any meaning (e.g., Bode et al., 2013). It is unknown, however, what processes underlie voluntary decisions in more complex, ecologically-valid environments, which require an ongoing, proactive commitment to an initially formed intention.

Studies investigating brain responses in the pre-decision period have found patterns of neural activity representing the upcoming choice, particularly when the choice is freely prepared (Libet, 1985; Soon et al., 2008, 2013; Bode et al., 2011; Leotti and Delgado, 2011). When participants were presented with random monetary gains, Leotti and Delgado (2011) found that reward circuitry was recruited in anticipation of a voluntary color choice but not preceding a forced choice. Predictive encoding of voluntary decisions has also been evident in areas of the sensory cortex (Kostelecki et al., 2012), frontopolar cortex and medial prefrontal cortex (Soon et al., 2008) when individuals chose randomly between left or right button presses (Soon et al., 2008; Kostelecki et al., 2012), or decided whether to add or subtract two numbers (Haynes et al., 2007; Soon et al., 2013). Furthermore, it was found that frontopolar cortex activity represented the content of simple decisions only if participants had the intention to voluntarily decide between two arbitrary options, but not if they believed themselves to be guessing (Bode et al., 2013). These studies suggest that voluntary decisions involve more proactive processes than instructed decisions, potentially recruiting different neural networks, depending on the nature of the decision.

Another noteworthy feature of previous studies that impacts their generalisability is that these studies have predominantly relied on participants making arbitrary choices, which were substantially removed from real-world situations. One side effect of this type of experimental design is that participants might have started producing relatively predictable sequences (Lages and Jaworska, 2012; Lages et al., 2013; Soon et al., 2014). In addition, some authors have argued that choices in some of these experiments barely qualify as true free decisions because they are irrelevant (Batthyany, 2009). In line with this reasoning, it has been argued that voluntary decisions should allow the freedom to choose between options “because of a desire, goal or preference” (Schall, 2001). This means there is a need for more ecological contexts in which measures can provide detail about the dynamic evolution of decision states (Smilek et al., 2006; Kingstone et al., 2008; Song and Nakayama, 2009; Pezzulo and Ognibene, 2012). In such scenarios, decisions can be conceptualized as more dynamic processes, rather than as single events in time. Hence, voluntary decisions might also be prone to change, because other choice options continue to be available.

Evidence of greater engagement of the frontoparietal network for voluntary decisions, irrespective of modality, has led some authors to propose this to be a higher order free decision network (Rowe et al., 2005; Thimm et al., 2012). The frontoparietal network is considered an attentional control network that extends across frontal and parietal cortices, including the anterior and dorsolateral prefrontal cortices, anterior cingulate cortex (ACC) and inferior parietal lobes (IPL; Vincent et al., 2008). Regions of this network have been commonly found to be more involved when people make free decisions than instructed decisions (Lau et al., 2004b; Coricelli et al., 2005; Rowe et al., 2005; Leotti and Delgado, 2011; Thimm et al., 2012). However, these studies have only reported activity at the time of response, when action selection is underway and there are demands on visual attention, which is also known to recruit the frontoparietal network (Scolari et al., 2015). In the context of a dynamic decision environment, it may be that regions for choice selection are proactively recruited during the entire decision period, leading up to the execution of the chosen option, while participants still engage with available choice options.

In order to investigate the proactive preparation of voluntary decisions, we created a novel decision-making paradigm in a virtual environment that was performed during functional Magnetic Resonance Imaging (fMRI). At the beginning of each trial, participants were asked to choose between three different colored doors at the end of a corridor, behind which they could find randomly hidden stars (functioning as reward cues). The stars solely provided motivation, as most real-world behavior involves some kind of reward, but participants were instructed that stars appeared randomly and no strategy was required. In the Instructed condition, participants were told the color of the door to choose, while in the Voluntary condition participants could freely choose between the colored doors. Participants then “walked” along the virtual corridor and selected their chosen door when they reached the end. We analyzed the period of time leading up to decision execution, conceptualizing the entire period as a dynamic decision with phases of choice selection and motor preparation.

Crucially, the doors were not visible when making the initial choice, as the location of each colored door only became visible at the halfway point in the corridor. Therefore, there could be no spatial attention or motor preparation differences between the different option doors during this initial phase of the trial. We predicted that activation of the frontoparietal network during the initial period would support its purported role in free choice selection. We also investigated whether we could decode the specific representation of the color door of choice. The second phase of the trial revealed the position of the colored doors and allowed participants to visually attend to their choices and begin specific motor preparation. This allowed us to decode the motor choice and, in addition, to investigate visual attention to the choices using eye-tracking. With this approach, we aimed to identify and explain differences in the representations of choice plans during proactive preparation of voluntary and instructed decisions.

## Materials and Methods

### Participants

Twenty-seven participants were recruited after providing written informed consent. All participants were right-handed, had normal or corrected to normal vision and no history of psychiatric or neurological trauma or disorder. Five participants were excluded due to excessive head movement. Data from the remaining 22 participants were used in the analyses (15 female, mean age 23 years, range 20–29 years). Participants were compensated monetarily for their time. This study was carried out in accordance with the recommendations of the National Statement on Ethical Conduct in Human Research, NHMRC Australia with written informed consent from all subjects. All subjects gave written informed consent in accordance with the Declaration of Helsinki. The protocol was approved by the University of Queensland Medical Research Ethics Committee.

### Experimental Task

**Figure 1:**
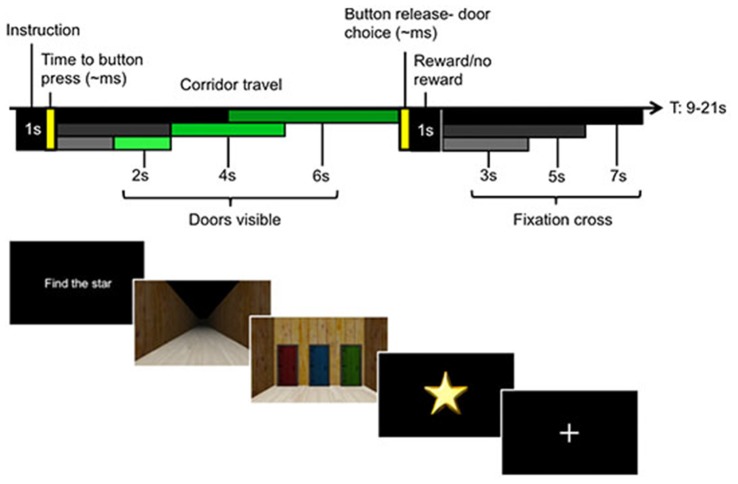
Experimental paradigm with single trial structure (upper panel) and participant screen views in the virtual environment (lower panel). Each trial began with an instruction screen. In Instructed trials, this told participants which color door to select, e.g., “Choose green”. In Voluntary trials, participants were told to “Find the star”, which allowed them to freely choose between either of the two colored doors they had not chosen on the previous trial. Instructed and Voluntary trials were interspersed to ensure that participants initiated a new decision on each trial. Participants were asked to make their choices at the start of the trial, before proceeding up a corridor. At the halfway point, the doors became visible and the position of each colored door became apparent. This enabled the participants to initiate motor preparation to choose the appropriate door at the end of the corridor. Every correct Instructed choice received a star, while stars were pseudorandomly gained in Voluntary trials. The upper left panel illustrates the different travel times to the halfway point (black to gray bars) and the travel times from halfway point to the door (dark to light green bars), equivalent to 2, 4, or 6 s. A fixation period separated each trial (jitter represented in black to gray bars).

Participants underwent a brief training session with instructions outside the scanner prior to completing the experimental task in the 3T MRI scanner. The task was based in a simple virtual environment, presented through Psychtoolbox on MATLAB (MathWorks Inc., Natick, MA, USA). The paradigm is shown in Figure 1. The virtual environment was used to create a dynamic task where participants were actively engaged in the epoch leading up to their decision execution. Participants viewed rendered images of the three-dimensional corridor with a first-person perspective from their current position, which was continuously updated in response to their movement. The task was to decide between three colors of doors, then to navigate a corridor until the doors were reached, and finally to select the door with the chosen color.

Each trial began with an instruction screen indicating one of two conditions. In the Instructed condition, participants were told which door color—red, green or blue—to choose (e.g., “Choose green”). Correctly choosing the instructed door always resulted in a star. The Voluntary condition asked participants to choose a color door of their own volition with the cue, “Find the star”. Choice outcomes were not directly indicated but had to be kept in memory. The participants were explicitly informed that the outcome for Voluntary trials was random, which allowed us to create incentive to make the choices while maintaining a scenario in which each choice was equally likely. Critically, the doors were not visible at the time participants were instructed to make their choices, so the choices were made for color and not for a particular spatial location.

The only restriction to the decision was that participants were instructed that they were not permitted to make the same color choice as in the previous trial (however, all choices were recorded and no error feedback was given if a choice repetition occurred). This had the advantage that it limited choice patterns, such as choice repetitions, that could occur with biases from the previous choice history (Bode et al., 2012; Lages and Jaworska, 2012). The second advantage was that we could assess the general task compliance by assessing how many of these unpermitted choice repetitions occurred. This design also ensured that participants actively made decisions at the start of each trial, relying on internal information to make their choice, and that this process was dissociated from both spatial attention and motor preparation.

Once a choice was made, participants traveled along the corridor (phase 1) by holding down a button with their left hand. At the half-way point in the corridor (phase 2), the doors at the end of the corridor became visible. Participants continued traveling down the corridor until they neared the end, where they could select one of the doors using three fingers of their right hand matched to the position of the three colored doors. The locations of each color door (left, middle, or right) were pseudorandomized each trial. This ensured that participants could only make their choice based on color information during phase 1 of the trial, while phase 2 permitted this color choice to then be mapped to the corresponding action. Importantly, this phase required only the mapping of the initial color choice to a door location. If the correct door was chosen in an Instructed trial, or if the rewarding door was selected in a Voluntary trial, a star was displayed as well as the total number of stars collected in the block. If no star was found, only the current count was displayed.

A fixation cross separated each trial. There was temporal jitter for the length of the corridor of approximately 4, 8 or 12 s, depending on the time participants made their door choice, as well as temporal jitter for the length of the fixation cross between trials (3, 5 or 7 s) to enable deconvolution of the hemeodynamic response function (HRF) between the different stages of the trial. There were five blocks of 36 trials each, consisting of equal numbers (6 trials) of Instructed/Voluntary × red/green/blue rewarded doors in a pseudorandomized order.

A brief questionnaire on task strategy was administered to participants at the end of the session outside the scanner, asking, “How motivated were you to find the stars?”, “Do you believe the stars were randomly hidden?”, “Did you use a strategy to find the stars?” and a blank space to detail any specific task strategy that participants might have used. Participants were also interviewed verbally to elaborate on any comments on their approach to the task.

Eye movements were measured using an infrared eye-tracking device (EyeLink 1000, SR Research Ltd., Kanata, ON, Canada). For one participant, data could not be collected for technical reasons. Fixations to the doors were registered for fixation events that corresponded to pixel regions for each of the three door locations. These pixel regions for each door were updated for consecutive 1 s screen viewing intervals, in order to account for changes in visual perspective while moving in the 3D environment. The duration of eye fixations to each of the doors was analyzed in separate time-bins over phase 2 for the Instructed and Voluntary conditions with a three-way ANOVA. Mauchly’s Test of Sphericity indicated that the assumption of sphericity had been violated (*p* < 0.001), so a Greenhouse-Geisser correction was applied.

### fMRI Acquisition

MRI volumes of the whole brain were acquired with a Siemens Trio 3T scanner (Erlangen, Germany) using a standard 32 channel head coil. For each of the five blocks, an average of 800–900 functional images (depending on speed of task completion) were acquired using a gradient-echo echo-planar imaging sequence (GE-EPI) with simultaneous multi-slice acquisition (multi-band slice acceleration factor = 4, 40 axial slices, TR = 700 ms, TE = 32.0 ms, FA 60°, FOV 192 × 192 mm, voxel size = 3 mm^2^, slice thickness = 3 mm with 10% slice gap). A high-resolution T1-weighted structural scan was acquired (TE = 2.32 ms, TR = 1900 ms, FA = 9°, FOV 192 × 192 mm, voxel size = 0.9 mm^3^). The first 12 s of scans were discarded to avoid magnetic saturation effects.

### Data Pre-Processing

All data was first preprocessed using SPM8 (Wellcome Department of Cognitive Neurology, University College London, London, UK). The images were spatially realigned using a six-parameter affine transformation to account for effects of head movement. The T1 structural image was coregistered to the mean of the realigned functional images and then spatially normalized to MNI space using the segment process of SPM8. These spatial normalization parameters were then applied to all functional images, which were then spatially smoothed with a Gaussian kernel of 8 mm full-width at half-maximum. For multi-voxel pattern analysis (MVPA), normalization and smoothing were not conducted at this stage (see below).

### Partial Least Squares Analysis

We conducted partial least squares (PLS) analysis to identify activity in brain regions that subserve Voluntary, as opposed to Instructed, decisions. This analytical technique is particularly utilized to extract key features from high-dimensional data and is robust to individual variations in activity. In this case, as we expected individual differences in the decision-making time (and strategy), this technique would best permit to identify core patterns of activity for Voluntary choices across all participants. This multivariate technique identifies patterns of voxel activity across the brain that covary with the experimental conditions (McIntosh et al., 1996), which results in a set of latent variables (LV), components that reflect patterns of brain activity related to the experimental conditions. The LVs are generated by singular value decomposition of a single matrix that contains all participants’ data, yielding a singular image of voxel saliences (i.e., a brain pattern reflecting task-related changes), a singular profile of task saliences (i.e., the degree to which each experimental condition contributes to the brain pattern), and a singular value (i.e., the amount of covariance accounted for by the LV). We conducted a mean-centering PLS analysis, using the onsets of the start of the corridor, separating correct trials only into Instructed and Voluntary conditions, for a period of 21 TR (corresponding to approximately 15 s of a hemodynamic response). Significance for each LV was calculated by a permutation test, which assesses that a singular value from permuted data (resampled 500 times) is larger than the obtained value (McIntosh et al., 1996). A second, independent step was a bootstrap estimation of the standard errors (resampled 100 times) to determine the reliability of the saliences for the brain voxels characterizing each pattern identified by the LVs (Efron and Tibshirani, 1986). A brain score was then calculated to represent the degree to which the pattern of brain activity was represented for each participant over each TR. Significance for the peak voxels was thresholded at a salience to standard error ratio >3, which is equivalent to *p* < 0.001 (Sampson et al., 1989). The regions reported showed activations across multiple time-points with a peak TR corresponding to approximately 8–10 s, which is displayed using Mango (Research Imaging Institute, University of Texas Health Science Center at San Antonio (UTHSCSA), TX, USA). Individual response function plots are displayed for main ROIs, calculated on BOLD percent signal change in a peak cluster of 27 voxels averaged over bilateral ROIs, and separated by condition.

### Multi-Voxel Pattern Analysis

MVPA was performed using a customized MVPA Toolbox, which has been applied in previous work (Bode et al., 2011, 2013), run in MATLAB in conjunction with SPM8. A general linear model was first estimated for each individual participant based on motion corrected, non-normalized, unsmoothed data to produce parameter estimates for each voxel in each run per participant across conditions. Beta images (regressors; condition × block + 6 motion correction parameters) were estimated for each participant in separate models, according to the type of decision category being examined. For the color-choice model, the estimated beta images corresponded to the start of the corridor (phase 1): Instructed/Voluntary × red/green/blue. For the position-choice model, the analysis of interest included beta images for the halfway point (phase 2): Instructed/Voluntary × left/middle/right. The same model, incorporating the location instead of color, was also run for phase 1 of the trial to check for evidence of potential planning unrelated to color. A separate set of regressors (of no interest) accounted for the errors (trials with incorrect responses, missing responses) in each model. Notable choice biases were observed in three participants: two demonstrated a strong color preference, and one demonstrated a strong position preference (fraction of selections differing by >10% above equal door selection). The color preferences resulted in unbalanced blocks where one of the colors was never chosen, so these participants were excluded from the MVPA color model analysis.

For each model, a three-way searchlight classification analysis with chance accuracy = 33.3% was conducted on the parameter estimates (beta images) for each participant separately (Kriegeskorte et al., 2006). Data for each condition and each run were extracted from a spherical cluster (3 voxel radius) centered in turn on each voxel in the brain, and subsequently transformed into pattern vectors (Soon et al., 2008). These vectors represented the spatial activation pattern for the respective experimental conditions from this particular position in the brain. A five-fold leave-one-out cross-validation procedure was implemented whereby a linear support vector machine classifier based on the LibSVM library (Chang and Lin, 2001) was trained on pattern vectors from all runs but one run, and then tested on the pattern vectors from the remaining run. In this way, data from each run was independently used as the test data set once while training on all other runs (see e.g., Bode et al., 2013). The average classification accuracy value for the respective cluster was then assigned to the central voxel. This procedure was repeated for each cluster in the brain to yield whole brain classification accuracy maps for each individual for each classification analysis of interest. To combine data across individuals, classification accuracy maps were registered to MNI space by applying the spatial normalization parameters extracted at the pre-processing stage of the univariate analyses, and were subsequently smoothed using an 8 mm FWHM Gaussian kernel. Second-level analysis was performed using SPM8. The images were entered into a one-sample *t*-test, with an explicit SPM brain mask, to identify voxels where classification was significantly above chance. Significance was determined at voxel-level *p* < 0.05, FWE-corrected. Pairwise *t*-tests were performed to compare peak decoding accuracy between conditions.

## Results

### Behavioral Task Performance

For the Instructed choice condition, mean performance, defined as correctly selecting the instructed colored door, was 97.2% (SD = 5.2%). For the Voluntary condition, correct performance was defined as choosing a color that was not the same color as chosen on the previous trial, and was 94.5% (SD = 4.7%). All participants but two reported being at least somewhat motivated to find the stars. Despite being instructed that the allocation of stars was random, 6/22 participants stated that they believed that outcomes were not random. Thirteen of 22 participants reported having used a strategy to choose the doors, and comments in the open response section indicated a perceived awareness of having produced some sequence history (14/22) to establish choice patterns. Comments in the open response section further indicated that the majority of participants (14/22) made color-based choices early in the trial, before they reached the halfway point. A small number (4/22) reported taking into account the position of doors to finalize their decision, and a further four participants reported not being aware of having used any strategy, and having acted randomly (Table 1). An overview of choice entropy, representing how distributed each participant’s choices were according to color and position, and the proportions of colors and positions chosen is provided in Supplementary Table S1 and Supplementary Figure S1.

**Table 1:** Summary of participant responses from questionnaire provided on task completion.

Questionnaire summary			
How motivated were you to find the stars?	Not at all	Somewhat	Very
	2	8	12
Do you believe the stars were randomly hidden?	Yes	No	
	16	6	
Did you use a strategy to find the stars?	Yes	No	
	9	13	
Open response: strategy	Sequential	Preference	None
	14	5	3
Open response: choice basis	Color	Position	Random
	14	4	4

### Duration of Eye Fixations

**Figure 2:**
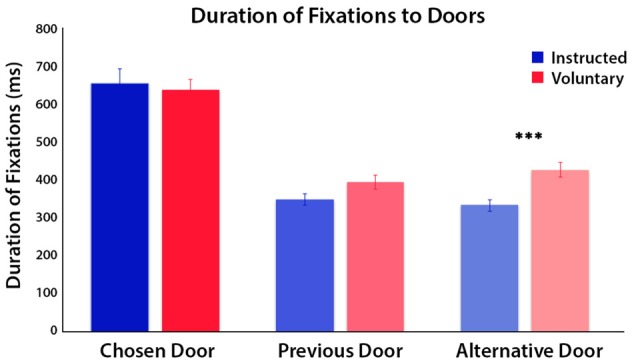
Mean duration of fixations on each of the doors for Instructed (blue) and Voluntary (red) decisions, during the second half of the trial when the doors were visible. Results of a three-way ANOVA reveal significant differences in fixation duration to the three doors between conditions (*p* < 0.001). The time spent fixating on the chosen door and previous door did not differ between conditions but time spent fixating on the alternative door, which was still available to choose, was greater in Voluntary trials (***p < 0.001).

A three-way ANOVA with factors door, time period and condition was conducted on the duration of fixations to the three doors. The factor of door was categorized as the chosen door, previous door (disallowed as it was chosen on the previous trial) and the alternative door for each condition, and was analyzed over the second phase of the trial when the doors were visible. The second trial phase was separated further into first and second half periods of time to examine if differences in fixations occurred only at the initial viewing or were maintained over time. We found a significant main effect of time period, indicating significantly longer fixation durations across all doors in the first half (*M* = 290 ms, SE = 13) compared to the second half of phase 2 (*M* = 176 ms, SE = 13; *F*_(1,20)_ = 28.60, *p* < 0.001). There was a significant main effect of condition, *F*_(1,20)_ = 21.80, *p* < 0.001 and a significant main effect of door choice, but also a significant interaction between door and condition, *F*_(1.65,33.05)_ = 4.82, *p* = 0.02 (Figure 2). *Post hoc* tests of this interaction effect were conducted using paired *t*-tests (Bonferroni-corrected) to compare Voluntary vs. Instructed conditions separately for each door. These showed a significantly longer duration of fixations to the alternative door in Voluntary decisions than in Instructed decisions, *t*_(20)_ = 5.07, *p* < 0.001. There was a trend towards a longer duration of fixations to the previous door in Voluntary trials than Instructed trials, but this did not survive Bonferroni correction, *t*_(20)_ = 2.29, *p* = 0.03. There was no significant difference in fixation durations to the chosen door between conditions, *t*_(20)_ = 0.64, *p* = 0.5. To check for anticipatory eye movements, we also examined the total number of fixations to the screen prior to the doors being visible and found only a trend, but no significant differences between conditions, *t*_(20)_ = 1.88, *p* = 0.07. There was also no significant difference in target fixation latency on the chosen door, once visible, between conditions, *t*_(20)_ = 1.22, *p* = 0.2.

### PLS Whole-Brain Networks

**Figure 3:**
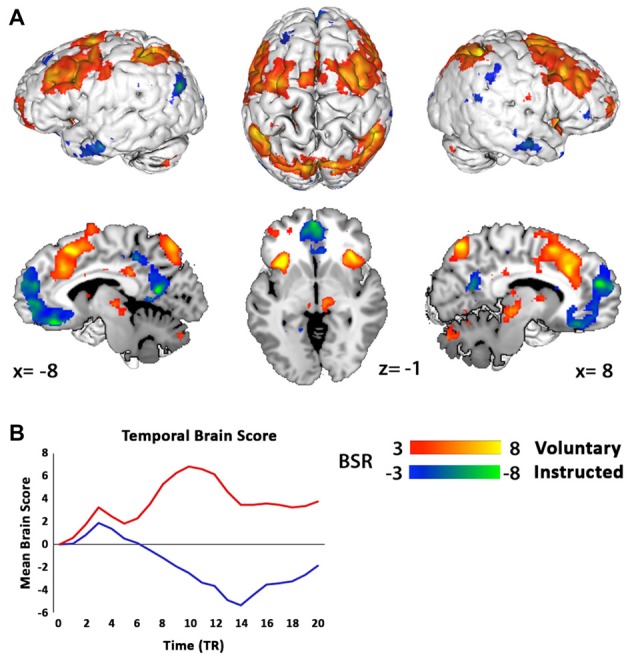
Partial least squares (PLS) analysis separates networks of regions where activations covaried with the onset of Voluntary decisions (red) and Instructed decisions (blue) at the start of the trial. **(A)** For Voluntary decisions, regions of the frontoparietal and salience networks were active, including the anterior cingulate cortex (ACC), supplementary motor area, bilateral dorsolateral prefrontal cortex (DLPFC), inferior parietal lobes (IPL) and insular cortices. Instructed decisions engaged a separate network, with activation predominantly in the medial prefrontal cortex. Results are thresholded at a bootstrap ratio (BSR) of 3 and displayed using Mango (Research Imaging Institute, UTHSCSA). **(B)** Mean brain scores of the LV1 pattern (*p* < 0.001) across the entire brain of each participant for Voluntary (red) and Instructed (blue) decisions over 21 TR scans (TR = 0.7 s; equivalent to 15 s of a hemeodynamic response) shows a functional differentiation between conditions. Note that the “negative” brain score here for Instructed decisions indicates that this network was dissociated from Voluntary decision activity, and does not represent deactivation.

The PLS analysis for two conditions at the start of the trial onset permitted only one LV, which was found to be significant (100% of model variance, *p* < 0.001), and differentiated Voluntary from Instructed decisions (Figures 3A,B). It is important to note that regions of activity for each condition reflect voxel activity that covaries with the onset of either a Voluntary or Instructed trial. Therefore, activity in the “positive” network identifies a whole-brain network that is more active for Voluntary decisions, whereas the “negative” network includes regions more active for Instructed decisions. In the Voluntary condition, there was widespread engagement of regions of the frontoparietal network, including the ACC, extending through to the supplementary motor areas (SMA), the dorsolateral prefrontal cortex (DLPFC), including the inferior and middle frontal gyri (IFG/MFG), and IPL. Activity in the bilateral insular cortices was also associated with Voluntary decision trials. Instructed trials were associated with a relative increase in activity in the medial prefrontal cortex and posterior cingulate cortex.

### MVPA Predictive Choice Patterns

**Figure 4:**
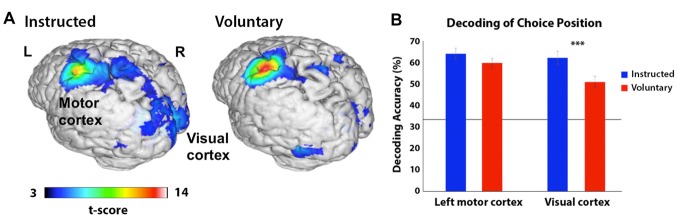
**(A)** Areas in which a searchlight analysis revealed significant decoding of the door position participants were preparing to choose (left, middle or right) in corridor task phase 2 (when the doors became visible). The *t*-score represents the group-level significance of decoding. Decoding accuracy was highest across conditions in the left motor cortex, but a second cluster in the visual cortex also contained discriminatory information (*p* < 0.05 FWE-corrected). Results are presented on a 3D-rendered standard brain template with Mango, thresholded at *p* < 0.001 for ease of viewing. **(B)** Decoding accuracy in the peak coordinates from **(A)** based on the position participants were preparing to select, separated by region and condition (Instructed in blue; Voluntary in red). The line represents chance accuracy (33%). In the left motor cortex, there is a trend towards higher decoding in the Instructed condition and in the visual cortex this difference is highly statistically significant (***p < 0.001). The horizontal line depicts chance accuracy (33%).

We analyzed the neural representation of color choice at both the start and halfway points of the trial but did not find significant clusters in either Voluntary or Instructed conditions. Sub-threshold clusters were, however, evident in the visual cortex at the start of the trial, at an uncorrected significance threshold (*p* < 0.001) with peak MNI coordinates at [−15 −79 10] when color conditions were combined across Voluntary and Instructed trials. No above-chance decoding of the door position was found at the start of the trial, confirming that participants were not relying on the position information available later in the trial to make their choices. At the halfway point, in both Instructed and Voluntary conditions, the choice corresponding to the position of the door could be decoded significantly above chance in a cluster including the left primary motor and somatosensory regions (Figure 4). The classifier accuracy was 64% for Instructed choice and 60% for Voluntary choice, corresponding to 31% and 27% above chance, respectively (peak MNI coordinates: Instructed [−42 −25 58], Voluntary [−42 −28 58], *p* < 0.05 FWE-corrected). A second cluster in the visual cortex also contained discriminatory information, with classification accuracy of 62% for Instructed choice and 51% for Voluntary choice (peak MNI coordinates: Instructed [9 −82 13], Voluntary [15 −82 16], *p* < 0.05 FWE-corrected). Decoding accuracy in the visual cortex was significantly higher in the Instructed condition compared to the Voluntary condition, *t*_(21)_ = 5.238, *p* < 0.001. Decoding accuracy in the motor cortex did not differ significantly between conditions but showed a similar trend, *t*_(21)_ = 1.828, *p* = 0.08.

## Discussion

Our findings reveal new insight into voluntary decision-making in a dynamic task environment, starting from initial intention to action execution. Using PLS, we show that regions corresponding to the frontoparietal and salience networks were activated when participants freely made their own choices rather than when they were instructed which door to choose. This activity was evident from the start of the trial, prior to any visual stimuli revealing the position of the doors ahead. Notably, this finding suggests that the neural activity during this proactive period reflected choice selection, a central process of voluntary decision-making, rather than any additional demands from attentional selection or motor preparation. Decoding of the specific color choice was not possible in either condition, but we found robust decoding of the motor choice from the time that the door positions became visible. During this later period of motor preparation, we also found that participants fixated longer on the alternative doors when their choice was freely made than when instructed. Together, these findings raise the possibility that proactive preparation of voluntary decisions engages ongoing demands on choice selection and maintenance, which might be more effortful for voluntary decisions as alternatives continue to be considered.

Results of this study provide support for a central role of the frontoparietal network in proactive voluntary choice selection. Regions of the frontoparietal network have been commonly associated with voluntary decisions, which require selection between multiple options (Lau et al., 2004b; Turk et al., 2004; Walton et al., 2004; Nachev et al., 2005; Rowe et al., 2005; Thimm et al., 2012). Thimm et al. (2012) proposed that the frontoparietal network may function as a free choice network that is recruited independent of modality, based on findings that perceptual and not only motor tasks recruited this network. Our study extends this proposal by finding recruitment of the frontoparietal network during a proactive decision-making period, separate from spatial attention processes or conflict between competing action plans, both of which are known to recruit the frontoparietal network (Scolari et al., 2015). This interpretation is also in line with other findings showing the involvement of the frontoparietal network when intentions had to be kept in memory before execution, in particular during the storage period (Gilbert, 2011; Gilbert et al., 2011). As in our study, the same network did not necessarily encode the outcome of the decision (Gilbert, 2011).

Frontoparietal activity has been found to scale with the amount of cognitive control required by tasks and, in particular, the amount of uncertainty within a decision (Fan, 2014; Fan et al., 2014). The common recruitment of this network during voluntary decisions is thought to result from the greater demand on selection processes required when choosing between multiple options, compared to simple goal maintenance for instructed choices (Frith et al., 1991; Hadland et al., 2001; Bunge et al., 2002; Leotti and Delgado, 2011; Thimm et al., 2012; Duncan, 2013). The DLPFC in particular has been suggested to be a central node of a higher order free choice network that is recruited to enable free selection among alternatives (Rowe et al., 2005; Thimm et al., 2012), both for rules and for actions (Rowe et al., 2008). However, in previous studies on DLPFC activation, it has not been possible to dissociate the effects of choice selection from the effects of motor processing (Curtis and D’Esposito, 2003). Our finding of DLPFC activity for voluntary mental color selection at a time preceding motor preparation provides good evidence for its role in free choice selection. The pre-SMA, another region, which has been commonly associated with voluntary choices across modalities (Cunnington et al., 2002, 2005; Lau et al., 2004a; Rowe et al., 2008; Taylor et al., 2008; Thimm et al., 2012), was also recruited for voluntary choice trials. In accordance with these previous studies, activity of the DLPFC and pre-SMA are indicative of choice selection occurring for voluntary decisions during this proactive period.

Furthermore, we found activity of the ACC, which has previously been found to be more activated for voluntary decisions than forced, or specified, decisions (Lau et al., 2004b; Walton et al., 2004; Forstmann et al., 2006). Some have suggested that activity in this region relates specifically to conflict between motor plans in related tasks (Thimm et al., 2012). However, recruitment of the ACC at a time preceding action preparation suggest a role related more broadly to information processing. One theory suggests that the ACC routes information on the basis of information content, or uncertainty, which in this case would relate to the higher number of options to compare and choose between in the voluntary trials compared to instructed trials (Fan, 2014). Another possibility is that ACC activation in this task may have been related to its role in conflict monitoring (Botvinick, 2007), as the restriction of choice options based on the previous choice required conflict resolution to inhibit the option that could not be chosen on that trial. Although conflict is arguably inevitable when choosing between highly similar options, as in most free choice tasks, there is some evidence that conflict and free choice selection engage different regions of the ACC (Nachev et al., 2005). Activation in our study cannot distinguish between the two.

The ACC together with the insula also forms a salience network responsible for detecting informative stimuli (Seeley et al., 2007; Menon and Uddin, 2010). The bilateral insula are a hub that integrate internal and perceptual inputs, which are then directed for attentional processing and goal-directed activity (Menon and Uddin, 2010). Similar to the ACC, the insula is also modulated by uncertainty in the expected choice outcomes (Critchley et al., 2001; Knutson and Greer, 2008; Mohr et al., 2010). Previous studies have found greater activation of this area for preference-based decisions over externally-dictated choices (Paulus and Frank, 2003; Turk et al., 2004). In addition to the anticipation of positive outcomes, the insula have been found to be activated preceding uncertain losses, which suggests that this network may be activated in arousal in general (Knutson and Greer, 2008). Our finding of salience network activity may be related to participants’ uncertain expectation of receiving a star from their choice. It has also been suggested that salience network activity is likely to be important during embodied decisions, where activity could enable switching between the various sources of information (Seeley et al., 2007). In our study, participants needed to attend to information related to color preference and value expectation as well as the upcoming visual cues in the virtual environment.

Although participant choices led to random reward outcomes, it is likely that the recent reward history influenced the expectations of the optimal choice for participants to make (Leotti and Delgado, 2011; Pezzulo and Ognibene, 2012; Haggard and Eitam, 2015). The tendency to see patterns in random events is well documented, and might have led to more systematic response patterns and strategies (Guth and Frankmann, 1965; Tricomi et al., 2004; Whitson and Galinsky, 2008; Yu and Cohen, 2008; Lages and Jaworska, 2012). We aimed to minimize these effects by disallowing repetition of the previous choice (Soon et al., 2008, 2014; Lages and Jaworska, 2012; Allefeld et al., 2013; Lages et al., 2013; Bode et al., 2014) and by clearly instructing participants that the distribution of stars was random. Nevertheless, a substantial proportion of participants suspected that the stars were not random, in line with previous reports that participants demonstrate the use of strategies when faced with random outcomes (Tricomi et al., 2004). Interestingly, these effects have been found to be enhanced when individuals perceive a lack of control (Whitson and Galinsky, 2008), which could have occurred in our task due to the inability to predict where the stars were hidden. It appears that even when individuals are aware that the environment is stochastic, given the freedom to choose, there is a tendency to track the statistical properties of the environment in order to try to optimize choice outcomes.

To what extent people engaged in such strategies was not directly investigated but the motivational value of finding the stars is likely to have differed between individuals in our study. The nature of the decision process and brain regions implicated might therefore have varied to some extent as well. Our use of PLS was designed to identify common networks activated, but it is possible that some individuals recruited a more extended decision-making network, engaging regions for strategic control or anticipatory reward processing. While these questions could be addressed in future work explicitly manipulating the probabilities of finding rewards or encouraging specific strategies, it should also be noted that in many real-life situations people likely employ individual strategies to guide their voluntary decisions.

With this study, we also sought to determine if it was possible to predict voluntary decisions in a more complex decision-making context. Surprisingly, we could not predict the decisions based on color choice at any point in the trial. This was true for voluntary as well as instructed trials, where participants necessarily had a color in mind. These results may be due to a lack of sensitivity in decoding, as subthreshold clusters in the visual cortex were evident at the start of the trial and color choices could be decoded (albeit at an uncorrected threshold) when voluntary and Instructed trials were analyzed together. Furthermore, the participants’ near-perfect accuracy in appropriately selecting one of the available color doors and the absence of position decoding in the first trial phase provide strong evidence that participants did indeed make initial decisions based on color. Once the doors became visible and initial color choices could be mapped onto positions to generate action choices, we found robust decoding for the position of the door participants were preparing to choose. The cluster in the left motor cortex most likely reflects preparatory motor plans differentiating between the fingers associated with selecting the left, middle or right doors. Finger-specific action plans have been reported in other modalities (Quandt et al., 2012). A second cluster in the visual cortex most likely indicates visual attention as participants fixated for longer on the door of choice.

In contrast to previous reports, we did not observe information predicting the decision outcomes in the frontoparietal network (Soon et al., 2008, 2013), nor in medial and lateral prefrontal cortex (Haynes et al., 2007), even though PLS analysis found this network to be highly activated for voluntary choices. It is possible that although these regions were involved in information processing, the content of the specific choices was encoded in separate regions. This dissociation between information encoding and retrieval has previously been reported for delayed intentions (Gilbert, 2011). However, it can be noted that, as the door associated with reward changed throughout the experiment, according to the pseudorandom receipt of stars, the only constant distinguishing features between doors were color and position. Higher-order decision factors may have been averaged across trials as the perception of the optimal door to choose changed. We also did not find frontopolar cortex activity to be predictive of decisions outcomes. It has been proposed that this region mediates cognitive branching between options that are held in the background (Koechlin and Hyafil, 2007), for example in a delayed intention (Gilbert, 2011) or sequential choice scenario (Soon et al., 2008). As we tried to minimize the reliance of choice on statistical tracking, with each trial requiring a new decision to be made after the cue, we expected less involvement of this region. Importantly, during the second task phase, higher-level planning areas may not have been predictive of the decisions, as participants were already able to transform their initial choices into action plans at this point.

Interestingly, we found that decoding was slightly lower in the motor cortex and significantly lower in the visual cortex for voluntary decisions as compared to instructed decisions. Patterns of eye fixations may provide some explanation for this finding. There was a systematic and significant difference in the duration of fixations between conditions as participants approached the doors: fixations to the chosen door were equal between conditions, but in voluntary trials the duration of fixations to the alternative available door throughout this period was longer. One possibility is that our neural findings may relate to a feature of voluntary decisions that would usually be overlooked in conventional, non-dynamic tasks: namely, individuals may continue to consider their available options throughout the time leading up to a decision. This freedom of choice could lead to more proactive visual exploration of the alternative option, as well as formation of a less stable choice representation, which may explain the weaker decoding of decision outcomes that we found in voluntary trials. Fixations to visual stimuli have been shown to influence upcoming decisions (Krajbich et al., 2010; Pezzulo and Ognibene, 2012), and ongoing consideration of the alternative door may reflect efforts to resolve uncertainty in deciding which door to select. However, it might also be that an inherent feature of voluntary decisions is to “keep one’s options open” (Klyubin et al., 2005).

In summary, we show that voluntary decisions were associated with activity of the frontoparietal network and salience network during a proactive decision period from intention formation to final action selection. We also find that the specific upcoming choice could be decoded from patterns of activity relating to motor preparation once the specific location of the selected door was known. This was evident in both motor and visual cortices in both conditions, although with lower decoding accuracy for voluntary decisions than the instructed decisions. Eye fixations further suggested that participants engaged more with the alternative door throughout the decision period when they were free to choose. These results suggest that free decisions recruit networks for choice selection in preparation for a decision, and that neural representations at this time reflect ongoing proactive processes relevant for decision-making and maintenance of voluntary decisions. Differences in engagement with the environment raise the possibility that during voluntary decisions individuals may hold choice options in a more active state during proactive preparation. Our study therefore constitutes an important step towards a better understanding of how decisions are made in more realistic ecological environments.

## Author Contributions

NR designed the study, collected data and performed analysis. SB contributed to study design and analysis. HB contributed to analysis. RC contributed to study design and interpretation. All authors contributed to manuscript preparation.

## Conflict of Interest Statement

The authors declare that the research was conducted in the absence of any commercial or financial relationships that could be construed as a potential conflict of interest. The reviewer C-TW and handling Editor declared their shared affiliation.
