# Supplementary material for: Proactive Recruitment of Frontoparietal and Salience Networks for Voluntary Decisions
Source: Front Hum Neurosci. 2017 Dec 12;11:610. doi: 10.3389/fnhum.2017.00610 (PMC5733024; doi:10.3389/fnhum.2017.00610)
Supplement: Supplementary file 1 [file Data_Sheet_1.docx]

Supplementary Material

Proactive recruitment of frontoparietal and salience networks for voluntary decisions

Rens, Natalie^1^, Bode, Stefan^2^, Burianová, Hana^3,4^, Cunnington, Ross^1,5^

*** Correspondence:** Ross Cunnington: r.cunnington@uq.edu.au

# Supplementary Data

Participant choice behaviour shows that overall there was well-balanced selection of the three doors according to colour, as well as position (Table 1). To further investigate choice behaviour, we calculated selection entropy (SE), which provides an indication of the randomness, or the sampling behaviour, of the choices made by participants (Zhang and Rowe, 2015). The SE provides a measure of the sampling over all available options over the period of time selected. The SE at each trial $i$ with a preceding window length $n$ is calculated according to:

$$SE\left( i \right)= H\left( Choice | Choice Set=\left\{ a,b,c \right\} \right)$$

$SE= - \sum_{\begin{aligned} k =\{a,b,c\} \\ m=\{1,2,3\} \end{aligned}} P\left( A_{j}=m, C_{j}=k \right) logP\left( A_{j}=m|C_{j}=k \right),(i-n+1)\leq j\leq i$

In this study, the choices were conditional on the previously chosen door, with three choice sets ($a,b,c$) corresponding to the unavailability of the red, green, or blue colour doors because the colour had been selected on the previous trial. The possible colour choices $m$ participants could make were red, green, or blue, with two responses available on each trial. For position choices, the choice set referred to the position of the door that corresponded to the unavailable colour. The possible position choices were the remaining two actions. We calculated SE for all three doors for the duration of the experiment ($n=90)$ for each participant (Figure 1). It was evident that two participants (S18, S19) had notable choice biases, which meant there was insufficient data on each run and their data was excluded from MVPA to avoid unbalancing the classifier training.

Table 1 Participant choice proportions, separated by door colour and position. In most cases, there was approximately even sampling, centred around 0.33 (three possible choices)

| Participant | Red | Blue | Green | Left | Middle | Right |
| --- | --- | --- | --- | --- | --- | --- |
| 1 | 0.32 | 0.33 | 0.35 | 0.34 | 0.36 | 0.30 |
| 2 | 0.31 | 0.32 | 0.37 | 0.28 | 0.44 | 0.28 |
| 3 | 0.32 | 0.32 | 0.36 | 0.31 | 0.34 | 0.34 |
| 4 | 0.34 | 0.33 | 0.33 | 0.26 | 0.33 | 0.41 |
| 5 | 0.36 | 0.32 | 0.32 | 0.27 | 0.36 | 0.37 |
| 6 | 0.34 | 0.32 | 0.34 | 0.31 | 0.36 | 0.33 |
| 7 | 0.31 | 0.33 | 0.36 | 0.31 | 0.34 | 0.35 |
| 8 | 0.33 | 0.34 | 0.32 | 0.32 | 0.40 | 0.28 |
| 9 | 0.32 | 0.32 | 0.36 | 0.31 | 0.35 | 0.34 |
| 10 | 0.33 | 0.33 | 0.34 | 0.32 | 0.37 | 0.31 |
| 11 | 0.33 | 0.33 | 0.34 | 0.35 | 0.33 | 0.32 |
| 12 | 0.34 | 0.35 | 0.31 | 0.28 | 0.32 | 0.40 |
| 13 | 0.34 | 0.33 | 0.32 | 0.28 | 0.36 | 0.36 |
| 14 | 0.29 | 0.38 | 0.33 | 0.31 | 0.38 | 0.31 |
| 15 | 0.33 | 0.34 | 0.33 | 0.33 | 0.28 | 0.39 |
| 16 | 0.38 | 0.30 | 0.32 | 0.31 | 0.41 | 0.29 |
| 17 | 0.33 | 0.30 | 0.37 | 0.36 | 0.31 | 0.34 |
| 18 | 0.43 | 0.38 | 0.19 | 0.32 | 0.34 | 0.34 |
| 19 | 0.22 | 0.37 | 0.42 | 0.28 | 0.36 | 0.36 |
| 20 | 0.36 | 0.34 | 0.30 | 0.28 | 0.37 | 0.35 |
| 21 | 0.34 | 0.32 | 0.33 | 0.33 | 0.34 | 0.33 |
| 22 | 0.36 | 0.29 | 0.36 | 0.33 | 0.39 | 0.27 |

Figure 1 Selection entropy for each participant, showing the distribution of choices across available options over the course of the experiment (perfect sampling = 1). Notable biases were observed in two participants (S18, S19), which meant exclusion from MVPA due to lack of sufficient choices to train the classifier.
